# Dietary Sucrose Determines Stress Resistance, Oxidative Damages, and Antioxidant Defense System in *Drosophila*

**DOI:** 10.1155/2022/7262342

**Published:** 2022-05-02

**Authors:** Olha Strilbytska, Tetiana Strutynska, Uliana Semaniuk, Nadia Burdyliyk, Volodymyr Bubalo, Oleh Lushchak

**Affiliations:** ^1^Department of Biochemistry and Biotechnology, Vasyl Stefanyk Precarpathian National University, 57 Shevchenka Str., Ivano-Frankivsk 76018, Ukraine; ^2^Laboratory of Experimental Toxicology and Mutagenesis, L.I. Medved's Research Center of Preventive Toxicology, Food and Chemical Safety, MHU, Kyiv, Ukraine; ^3^Research and Development University, Ivano-Frankivsk, Ukraine

## Abstract

Varied nutritional interventions affect lifespan and metabolic health. Abundant experimental evidence indicates that the carbohydrate restriction in the diet induces changes to support long-lived phenotypes. Reactive oxygen species (ROS) are among the main mechanisms that mediate the effect of nutrient consumption on the aging process. Here, we tested the influence of sucrose concentration in the diet on stress resistance, antioxidant defense systems, and oxidative stress markers in *D. melanogaster.* We found that high sucrose concentration in the fly medium leads to enhanced resistance to starvation, oxidative, heat, and cold stresses. However, flies that were raised on low sucrose food displayed increased levels of low-molecular-mass thiols, lipid peroxides in females, and higher activity of antioxidant enzymes, indicating that the consumption of a low carbohydrate diet could induce oxidative stress in the fruit fly. We found that the consumption of sucrose-enriched diet increased protein carbonyl level, which may indicate about the activation of glycation processes. The results highlight a strong dependence of oxidative metabolism in *D. melanogaster* from dietary carbohydrates.

## 1. Introduction

Dietary carbohydrates play a key role in human nutrition by altering many vital functions. Carbohydrates are the predominant sources of energy to maintain processes in the human body and organismal homeostasis in general. The availability of carbohydrates influences metabolic and signaling pathways [1], energy metabolism [2], and, in turn, organismal aging.

Despite the beneficial role of carbohydrates, their consumption can cause negative effects, depending on the type and dosage [3]. A high carbohydrate diet often leads to metabolic disorders, including insulin resistance [4]. The consumption of diets with high carbohydrate content may result in hyperglycemia, which is associated with the formation of glycated products [5]. These events trigger free radical overproduction and antioxidant defense impairment, leading to cell damage and disease progression [6, 7]. Consequently, carbohydrates can induce reactive oxygen species production indirectly via modified biomolecules [8].

The fruit fly *Drosophila melanogaster* is a well-suited model organism to investigate the effects of nutrient composition on physiological and biochemical parameters [9]. In addition to short lifespan, short generation time, and high fecundity [9], *Drosophila* is characterized by conserved mechanisms, which are similar to those in mammals [10]. Thus, *Drosophila* is a popular model organism for nutrigenomic studies [11]. Using *Drosophila* as a model, it was demonstrated that low carbohydrate consumption was related to mild oxidative stress development [12]. It was also shown that ROS production during carbohydrate restriction was required for lifespan extension in *D. melanogaster* [12], which is associated with the activation of antioxidant defense systems via the development of moderate oxidative stress [13]. In particular, low-molecular mass antioxidants, antioxidant enzymes, and uric acid levels were increased under a low carbohydrate diet [14]. Hence, carbohydrate content in the diet can lead to a shift in oxidative metabolism.

In our previous work, we have investigated metabolic changes caused by a diet with different sucrose content. It was shown that the macronutrient ratio contributed to the aging process and enzyme activities, which were related to central metabolic pathway functioning [15]. Therefore, in the current study, we aimed to investigate the influence of sucrose concentration in the diet on stress resistance, antioxidant defense systems, and oxidative stress markers in *D. melanogaster*. To address these questions, we reared individuals on the four diets containing constant yeast concentration and increasing amounts of sugar, ranging from 1% to 20%. The detected experimental outcomes were as follows: (1) increased survival under starvation, oxidative stress, heat, and cold stresses were caused by high sucrose concentration in the fly diet, (2) the consumption of low carbohydrate diets led to higher levels of low-molecule mass thiols, lipid peroxides in females, and higher activity of antioxidant enzymes, and (3) high sugar diet resulted in increased protein carbonyls level. Thus, our data indicated that sucrose consumption affected stress resistance and oxidative metabolism in *D. melanogaster.*

## 2. Materials and Methods

### 2.1. Reagent

Nipagin, agar, menadione, agarose, phenylmethylsulfonyl fluoride (PMSF), reduced glutathione (GSH), NADP+, NADPH, glucose-6-phosphate, ethylenediaminetetraacetic acid (EDTA), xylenol orange, cumene hydroperoxide, 2,4-dinitrophenylhydrazine (DNPH), N,N,N',N'-tetramethylethylenediamine (TEMED), quercetin, 1-chloro-2,4-dinitrobenzene (CDNB), Tris-HCl, and 5,5′-dithio-bis (2-nitro) benzoic acid (DTNB), were purchased from Sigma-Aldrich Chemie GmbH (Germany). Sucrose was purchased from TM Fluka. All other reagents were of the analytical grade from local suppliers (Ukraine).

### 2.2. Flies and Experimental Design

We used *Сanton-S* [*D. melanogaster* Meigen] flies received from the Bloomington Stock Center (Indiana University, USA). Flies were kept at 25°C, 60–70% relative humidity, and a photoperiod 12 h day/night. Regular food was composed of 4% of sucrose (TM Fluka), 4% of dry yeast (type “Extra”, TM “Lvivski drizhdzhi”), 1.2% of agar and 0.18% napagin, which possesses antibacterial and antifungal properties. Four-day-old adult flies were separated by sex under carbon dioxide anesthesia and kept for one more day on the medium for recovery. Then, five-day-old flies were transferred into 1.5 L demographic cages (at a standard density of 200 flies per cage), with an attached vial containing 5 ml of an experimental medium composed of 4% of dry yeast and sucrose in different concentrations: 1, 4, 10, or 20%, 1.2% of agar, and 0.18% of nipagin. Food was changed every other day. Flies were kept for 25 days and used for physiological tests or frozen in liquid nitrogen for the measurements of biochemical parameters.

### 2.3. Resistance toward Starvation and Oxidative Stress

Sensitivity to oxidative stress was assessed by adding 20 mM menadione as an inducer of oxidative stress [16]. Fifteen flies of particular sex and cohorts were transferred into 15 ml empty vials for 2 h starvation. Next, flies were transferred into vials containing folded and rammed strips (2.4 × 12 cm) of 4-layer cellulose filter paper soaked with 0.8 ml of 20 mM menadione in a 5% sucrose solution [16]. Starvation resistance was measured in flies that were given 0.5% agarose. Vials were changed every other day, and the number of dead flies was recorded every 6/12/6 hours (at 9:00 a.m., 3:00 p.m. and 9:00 p.m.) for menadione and starvation experiments until the last fly died.

### 2.4. Heat and Cold Tolerance Assays

After 25 days of consumption of the medium with different sucrose content, flies were exposed to heat and cold stress. To modulate heat stress, single flies of particular sex were placed into small glass vials covered by a cotton plug, which then were put into a water bath at 43°C [17]. Full immobility was considered to be coma occurring, and time was recorded. Next, paralyzed vials were removed from the water bath to room temperature (21–23°C), and the time needed for recovery was scored. Ten flies of a particular sex and experimental cohort were tested for the determination of heat tolerance. Cold-induced coma was performed by placing a vial with 10 flies of each group on ice in the isolated boxes (0°C) for 1 minute [18]. Recovery time that is usually referred to as “chill coma recovery” was recorded [19]. Then, motionless flies were transferred into Petri dishes at room temperature (21–23°C), and the time of recovery was recorded.

### 2.5. Activities of Antioxidant and Associated Enzymes

Weighed flies were homogenized (1 : 10 w/v) in buffer medium, which consisted of 50 mM potassium phosphate buffer (pH = 7.5), 1 mM PMSF, and 0.5 mM EDTA. Flies were crashed using a Potter-Elvejhem glass homogenizer. Enzymatic activities in fly extracts were measured as was described previously [15]. The activity of superoxide dismutase (SOD, EC 1.15.1.1) was determined spectrophotometrically by detecting quercetin oxidation inhibition by superoxide anion at 406 nm using a Spekol 211 spectrophotometer (Carl Zeiss, Jena, Germany). The reaction mixture contained 30 mM Tris buffer (pH 10.0), 0.5 mM EDTA, 0.8 mM N,N,N′,N′-tetramethylethylenediamine, 0.05 mM quercetin, and 1–100 *μ*l of supernatant [20]. One unit of SOD activity is determined as the amount of enzyme quantified per milligram of protein, which inhibits the quercetin oxidation reaction by 50%. Catalase (EC 1.11.1.6) activity was assayed spectrophotometrically at 240 nm by detecting the decomposition of hydrogen peroxide. The reaction mixture for catalase activity assay contained 50 mM KPi (pH 7.0), 0.5 mM EDTA, 10 mM H_2_O_2_, and 2 *μ*l of supernatant. A molar absorption coefficient of hydrogen peroxide 39.4 M^−1^·cm^−1^ was used to calculate catalase activity.

The activity of glutathione-*S*-transferase (GST, EC 2.5.1.18) was measured by the monitoring of absorbance of the conjugate formed between glutathione and CDNB [21]. The reaction mixture for GST activity assay contained 50 mM KPi (pH 7.0), 0.5 mM EDTA, 5 mM glutathione (GSH), 1mM CDNB, and 10 *μ*l of supernatant. Changes in absorbance were detected using a Spekol 211 spectrophotometer at 340 nm, and GST activity was calculated using the molar absorption coefficient for CDNB 9600 M^−1^·cm^−1^.

Glucose-6-phosphate dehydrogenase (G6PDH, EC 1.1.1.49) and isocitrate dehydrogenase (IDH, EC1.1.1.49) activities were determined as described previously [7]. The rates of NADPH production were determined by measuring the change in absorbance at 340 nm. The reaction mixture for G6PDH activity assay contained 50 mM KPi (pH 7.0), 0.5 mM EDTA, 5 mM MgCl_2_, 2 mM glucose-6-phosphate, 0.2 mM NADP, and 25 *μ*l of supernatant. The activity of IDH was determined using reaction mixture containing 50 mM KPi (pH 7.0), 0.5 mM EDTA, 0.5 mM isocitrate, 0.2 mM NADP, and 25 *μ*l of supernatant. To calculate enzyme activities, an absorption coefficient for NADPH 6220 M^−1^·cm^−1^ was used.

Enzyme activities were presented as international units per milligram of soluble protein. Protein concentration was measured using the Bradford method [22]. The method is based on the formation of a complex between Coomassie Brilliant Blue G-250 and proteins in supernatant. Bovine serum albumin was used as a standard.

### 2.6. Assays of Protein (P-SH) and Low Molecular Mass Thiols (L-SH)

Free thiols were measured spectrophotometrically by registering the absorption of thiol conjugates with DTNB at 412 nm. The total thiol content (the sum of protein and low-molecular-mass thiol-containing compounds) was measured in the supernatants obtained after 30 mins of incubation of the supernatant with DTNB and potassium phosphate buffer (pH = 8.0). The low-molecular mass thiol level was determined by adding 30% trichloroacetic acid (TCA) to supernatants with further centrifugation for 5 min, 4°C, at 13000*g* to remove precipitated protein. The resulting supernatants were then incubated for 30 min with DTNB and Tris-HCl buffer (pH = 9.0). The low-molecular mass thiol content was measured using a Spekol 211 spectrophotometer at 412 nm. An extinction coefficient of 14 × 10^3^ M^−1^·cm^−1^ was used for calculations. The protein thiol level was calculated by subtracting the low-molecular thiol content from the total thiol content. Low-molecular thiol concentration was presented as the micromoles of SH-groups per gram of fly wet weight, and high-molecular thiol concentration was expressed as the nanomoles of SH-groups per milligram of soluble protein.

### 2.7. Determination of Protein Carbonyl and Lipid Peroxide Levels

Protein carbonyl groups (PC) were determined by measuring the amount of 2,4-dinitrophenylhydrazone created in the reaction with 2,4-Dinitrophenylhydrazine (DNPH) as described previously [7]. Detection was carried out spectrophotometrically at 370 nm and calculated using the extinction coefficient of 22 mM^−1^·cm^−1^. Results were presented as nanomoles per milligram of protein. The lipid peroxide (LOOH) level was determined using xylenol orange [23]. For this, flies were homogenized (1 : 20 (w:v)) in 96% cold (near 5°C) ethanol with subsequent centrifugation for 5 min, 4°C, at 13000*g*. The obtained supernatants were used for lipid peroxide content determination. The data were presented as nanomoles of cumene hydroperoxide equivalents per gram of fly weight [7].

### 2.8. Statistical Analysis and Graphical Representation

Data are presented as mean ± SEM. Differences between the groups were considered significant at *p* < 0.05. All calculations and graphs were made using “Prism” (Prism GraphPad Software, version#6). All datasets were normally distributed and their variances were homogenous. The normality of distribution was evaluated by the Shapiro–Wilk test, whereas the homogeneity of variances was checked by the Levene test, implemented in GraphPad Prism. Two-way ANOVA, followed by Tukey's test, has been used to find the differences between the groups. Survival curves were analyzed by the log-rank (Mantel–Cox) test.

## 3. Results

### 3.1. Dietary Sucrose Affects *Drosophila* Stress Resistance

**Figure 1:**
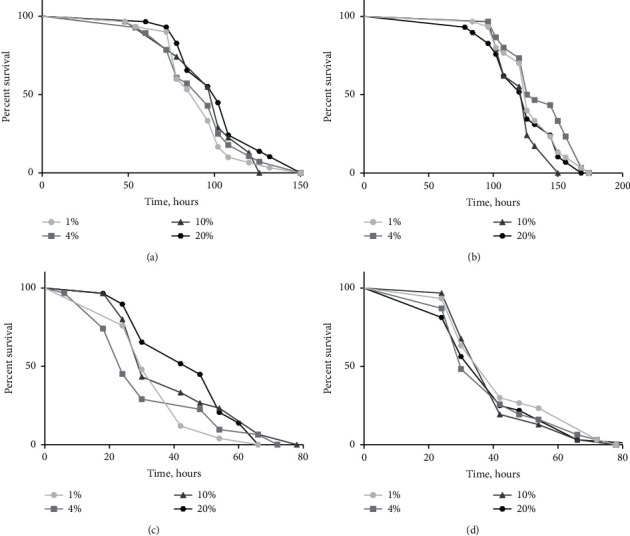
Resistance to starvation in males (a) and females (b), as well as oxidative stress resistance in males (c) and females (d). Each curve shows the fraction of individuals alive as a function of time with approximately 50 flies per group. The statistical analysis of differences in survival was conducted with a log-rank test.

To study the effect of dietary sucrose on stress resistance, flies were subjected to starvation condition. Resistance toward starvation in males was higher under the consumption of 20% of sucrose as compared to 4% (log-rank test, *p*=0.042; *χ*^2^ = 4.144) (Figure 1(a)). We observed increased survival under starvation conditions in females, which consumed the medium with 10% (log-rank test, *p*=0.003; *χ*^2^ = 9.105) and 20% (log-rank test, *p*=0.026; *χ*^2^ = 4.929) of sucrose as compared to 4% (Figure 1(b)).

To further investigate the role of dietary sucrose on the stress resistance, we treated flies with 20 mM menadione, which is shown to induce oxidative stress conditions. Males reared on the medium with 20% of sucrose were more resistant to oxidative stress as compare to those fed with 1% sucrose diet (log-rank test, *p*=0.042; *χ*^2^ = 4.144) (Figure 1(c)). However, the consumption of sucrose at different concentrations had no impact on oxidative stress resistance in females (Figure 1(d)). Thus, a higher concentration of sucrose in diet enhances a response toward starvation in flies of both sex and oxidative stress in males.

**Figure 2:**
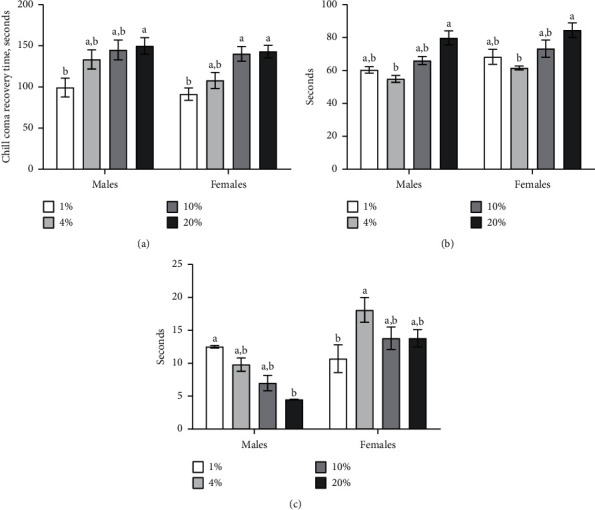
Sensitivity toward cold (a) and heat-induced stress ((b time required to full fly immobility and referred as heat paralysis; (c) time needed for the full recovery of locomotion). Results are presented as mean ± SEM, *n* = 10. Values were compared by Tukey's test: a indicates the highest values among all tested groups, and b indicates significant difference from *a,* with *p* < 0.05.

To investigate the influence of sucrose concentration in the diet on *Drosophila* cold tolerance, we determined the time needed to recover from chill coma after the exposure of experimental flies to 0°C during 1 min, which is referred to as chill coma recovery time (CCRT). Resistance to cold stress depended only on the sucrose content (ANOVA: *F*_*3,16*_ = 11.11, *p*=0.0003), however, it did not depend on fly sex and interaction of both factors (Table 1). Males which consumed the medium with 1% of sucrose recovered 34% faster as compared to 20% of sucrose (Figure 2(a); Tukey's test, *p*=0.040). We observed that females that were fed with 10 and 20% of sucrose recovered, respectively, 31% and 36% faster as compared to 1% of sucrose (Figure 2(a); Tukey's test, *p*=0.026, *p*=0.014, respectively). Moreover, we detected a negative dependence of recovery after chill exposure in flies of both sexes with sucrose content in the diet. We concluded that high sucrose diet enhanced cold resistance in *D. melanogaster.*

The tolerance toward heat exposure in flies was associated with fly sex (ANOVA: *F*_*1,16*_ = 68.67, *p*=0.019) and sucrose concentration (ANOVA: *F*_*3,16*_ = 16.27, *p* < 0.0001) (Table 1). Males, which consumed medium with 20% of sucrose, entered heat coma by 31% slowly as compared to the group with 4% of sucrose in diet (Figure 2(b); Tukey's test, *p* < 0.003). The recovery from heat coma was 24% faster in males fed with 20% sucrose diet as compared to 1% sucrose (Figure 2(c); Tukey's test, *p*=0.004). Interestingly, similar effects were observed in females. Indeed, 20% sucrose in the medium slowed the entrance to heat coma by 27% in females compared to 4% (Figure 2(b); Tukey's test, *p* < 0.006). Moreover, recovery after heat coma induction was 41% faster in females that were reared on the medium with 1% of sucrose as compared to 4% (Figure 2(c); Tukey's test, *p*=0.025).

### 3.2. Activities of the First-Line Antioxidant Enzymes Depend on Sucrose Concentration in the Diet

**Figure 3:**
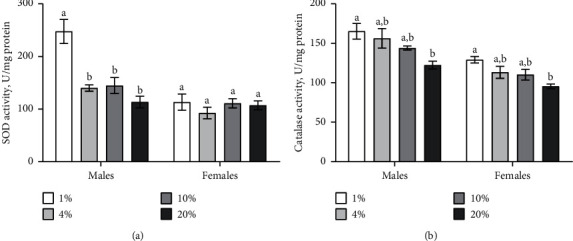
Activities of SOD and catalase in flies that consumed diets with different sucrose concentrations. Results are shown as mean ± SEM, *n* = 4. Values were compared using Tukey's test: a indicates the highest mean among all tested groups; b indicates a significant difference from a, with *p* < 0.05.

SOD activity in flies significantly depended on sucrose concentration in diet (ANOVA: *F*_*3,24*_ = 11.62, *p* < 0.0001), sex (ANOVA: *F*_*1,24*_ = 34.94, *p* < 0.0001) and interaction between sex and diet (ANOVA: *F*_*3,24*_ = 8.718, *p* < 0.0004) (Table 1). The activity of SOD was higher in males, which consumed medium with 1% of sucrose as compared to all the rest of the experimental groups, i.e., 4% (by 42%), 10% (by 43%), and 20% (by 54%) (Figure 3(a); Tukey's test, *p*=0.0002, *p*=0.0003; *p* < 0.0001, respectively). However, we did not detect the dependence of SOD activity in females from sucrose concentrations the diet (Figure 3(a)).

The activity of catalase was associated with sex (ANOVA: *F*_*1,24*_ = 47.36, *p* < 0.0001) and sucrose concentration (ANOVA: *F*_*3,24*_ = 9.977, *p*=0.0002) (Table 1). The consumption of the medium with 1% of sucrose lead to 25% higher catalase activity in males as compared with those fed with a diet of 20% of sucrose (Figure 3(b); Tukey's test, *p*=0.061). Females reared on the medium with 1% of sucrose displayed 26% enhanced catalase activity as compared to those that were reared on the medium with 20% of sucrose (Figure 3(b); Tukey's test, *p*=0.049). Hence, we can suggest that low sucrose concentration in the diet can lead to the development of moderate oxidative stress in fruit flies.

### 3.3. Activities of the Second-Line Antioxidant Enzymes Depend on Sucrose Concentration in the Diet

**Figure 4:**
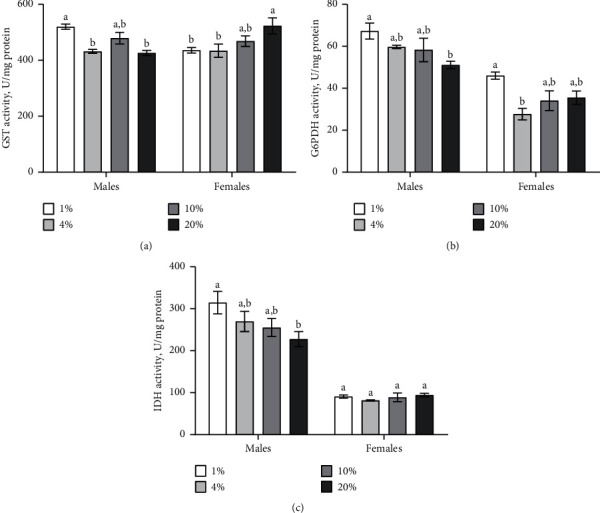
Effect of dietary sucrose in the diet on the activities of GST, NADP-dependent G6PDH, and IDH. Results are shown as mean ± SEM, *n* = 4. Values were compared by Tukey's test: a indicates the highest mean among all tested groups; b indicates a significant difference from a with *p* < 0.05.

G*S*T activity was determined by the interaction between the fly sex and sucrose concentration in the diet (ANOVA: *F*_*3,24*_ = 8.961, *p*=0.0004) (Table 1). We found increased G*S*T activity by 18% in males, which consumed food with 1% of sucrose as compared to 4 and 20% of sucrose in diet (Figure 4(a); Tukey's test, *p*=0.030; *p*=0.018, respectively). However, we observed an opposite response in female flies. The consumption of medium with 20% of sucrose caused 18% higher GST activity in females as compared to 1% and 4% of sucrose (Figure 4(a); Tukey's test, *p*=0.032; *p*=0.028, respectively).

G6PDH activity in flies strongly depended on the concentration of sucrose in the diet (ANOVA: *F*_*3,24*_ = 6.762, *p*=0.001) and sex (ANOVA: *F*_*1,24*_ = 93.86, *p* < 0.0001) (Table 1). We found 47% higher G6PDH activity in males reared on the medium with 1% of sucrose as compared to 20% (Figure 4(b); Tukey's test, *p*=0.045). We also observed increase higher G6PDH activity by 40% in females under the consumption of medium with 1% of sucrose as of compared to 4% (Figure 4(b); Tukey's test, *p*=0.016).

IDH activity in flies significantly depended on fly sex (ANOVA: *F*_*1,24*_ = 128.3, *p* < 0.0001) (Table 1). The consumption of diet with 1% of sucrose caused 28% higher activity of IDH as compared to 20% of sucrose in the diet (Figure 4(c); Tukey's test, *p*=0.021). Interestingly, IDH activity in females was not affected by dietary sucrose (Figure 4(c)).

### 3.4. Oxidative Stress Markers Depend on Sucrose Concentration in the Diet

The determination of free radical process perturbations includes the evaluation of oxidative stress markers. Those markers are the certain products of ROS interaction with varied biomolecules [24]. Proteins, lipids, DNA, and carbohydrates are the susceptible targets for ROS [5]. Enchased lipid oxidation is reflected by the increased formation of lipid peroxide groups [25]. Protein modification by ROS, usually, involves the emergence of additional carbonyl groups, which are one of the most used fingerprints of protein modification by oxidation [20]. Another important hallmark of oxidative stress is the level of reduced thiol groups of protein amino acids. Protein cysteine thiol groups are very sensitive to changes in a cellular redox state [26]. Under shifts in redox balance, protein thiols are oxidized first of all. Glutathione represents a significant fraction of low-molecular weight compounds, containing sulfhydryl groups. Thus, its interaction with ROS might protect protein thiol groups [25].

**Figure 5:**
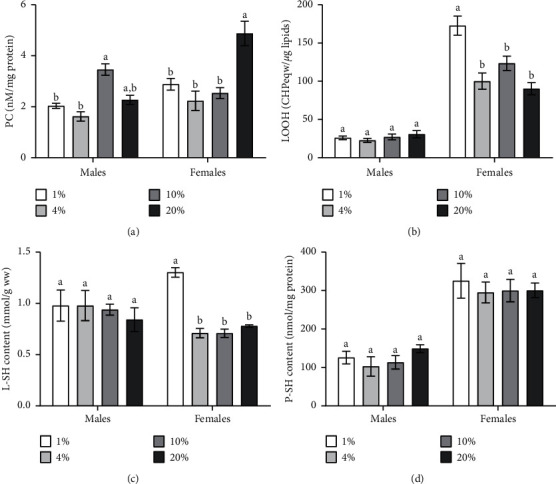
Contents of carbonyl protein, lipid peroxide, low-molecular mass, and protein thiol groups in 25-day-old adult *D. melanogaster* that are fed with diets containing different sucrose concentrations. Results are shown as mean ± SEM, *n* = 4. Values were compared by Tukey's test: a indicates the highest mean among all tested groups; b indicates a significant difference from a with *p* < 0.05. CHP, cumene hydroperoxide.

Protein carbonyl groups were associated with sucrose concentration (ANOVA: *F*_*3,16*_ = 13.33, *p*=0.0001), sex (ANOVA: *F*_*1,16*_ = 16.40, *p*=0.0009), and the interaction between these factors (ANOVA: *F*_*3,16*_ = 13.87, *p* < 0.0001) (Table 1). Higher protein carbonyl levels by 41% and 47% were found in males fed with a diet of 10% of sucrose as compared to 1% and 4% of sucrose (Figure 5(a); Tukey's test, *p*=0.034; *p*=0.004, respectively). The consumption of 20% of sucrose caused higher protein carbonyl content in females as compared to diet with 1% (by 41%), 4% (by 54%), and 10% (by 48%) of sucrose (Figure 5(a); Tukey's test, *p*=0.002).

Lipid peroxide content was dependent on sex (ANOVA: *F*_*1,16*_ = 306.3, *p* < 0.0001), sucrose concentration (ANOVA: *F*_*3,16*_ = 11.17, *p*=0.0003), and their interaction (ANOVA: *F*_*3,16*_ = 12.00, *p*=0.0002) (Table 1). Female flies fed with a diet of 1% of sucrose had higher lipid peroxides as compared to the rest of the experimental diets (Figure 5(b)). Thus, low sucrose content may stimulate lipid peroxidation in females. No significant difference in lipid peroxide content was observed in males fed with diets containing different sucrose concentrations (Figure 5(b)).

The content of low-molecular compounds with thiol groups (L-SH) was affected by sucrose concentration in the diet (ANOVA: *F*_*3,24*_ = 5.992, *p*=0.003) and the interaction of sex and diet (ANOVA: *F*_*3,24*_ = 4.414, *p*=0.013) (Table 1). Females fed with a diet of 1% of sucrose had 46% higher L-SH content as compared to 4% and 10% of sucrose. The consumption of diet with 1% of sucrose by females also caused 40% higher L-SH content as compared to 20% group (Figure 5(c); Tukey's test, *p*=0.0092). Hence, higher L-SH was associated with lower sucrose concentration in the diet of females. Thiol groups in proteins (P-SH) were dependent on sex (ANOVA: *F*_*1,16*_ = 100.5, *p* < 0.0001) (Table 1). There was no significant difference in the concentration of P-SH in response to dietary sucrose in the flies of both sexes (Figure 5(d)).

## 4. Discussion

Dietary conditions influence varied traits, supporting organism functionality and homeostasis [27]. Nutrition determines cellular metabolism and signaling and influences gene expression profiles and epigenetic signatures [28]. However, a shift in dietary conditions might cause negative consequences, such as oxidative stress because of an increase in ROS generation and/or decreased detoxification. Under optimal conditions, ROS are involved in normal cellular communication and immune response. However, if the processes of its generation and elimination are disrupted, an oxidative stress is observed [5, 29].

We have previously shown the transgenerational influence that specific dietary macronutrients provided in the parental diet, namely carbohydrates, can have on the next generation, influencing enzyme activities, metabolic pathways, and antioxidant defenses in progeny *Drosophila* [30]. In current work, we investigated the influence of sucrose content in the diet on stress resistance, antioxidant defense system, and free radical processes. We observed that higher sucrose concentration in the food is related to increased resistance toward starvation in flies of both sexes and oxidative stress resistance in males (Figure 1(a), 1(b)). Possibly, the increased storage of nutrients depends on food preferences based on taste. Thus, a high sucrose diet may result in an increase in calorie intake [31]. Our data are in good agreement with the number of studies supporting the fact that the consumption of high carbohydrate diet increases resistance to starvation conditions [32, 33]. We also demonstrated that sucrose-enriched diet increased resistance toward oxidative stress (Figure 1(c)). There are conditions in which dietary carbohydrates could activate antioxidant defense systems without causing oxidative damage. It means they behave as antioxidants [7]. Glucose is essential for the functioning of the pentose phosphate pathway (PPP), which generates the reducing equivalents in the form of NADPH. The major functions of NADPH are the reduction in oxidized glutathione and protein thiols, the synthesis of lipids and DNA, and xenobiotic detoxification [34].

We also found that the consumption of sucrose-enriched diet increased resistance to the exposure of high temperatures. We observed that high sucrose diet increased the time needed for heat coma entrance and shortened heat coma recovery in males. It suggests increased resistance to heat under the consumption of diet with high sucrose content. Carbohydrate-enriched diet was shown to be associated with obesity because of the excessive storage of dietary energy in fruit fly fat body [35]. The role of fat bodies during stress conditions is important because they serve as an energy source that the cell needs redirect to maintain macromolecule repairs in response to stress-inducible damages [36].

It was demonstrated that thermal stress causes a decrease in *Drosophila* fat reserves [37]. Klepsatel and colleagues have suggested that the body fat storage declines under heat exposure, probably, because of the apoptosis of fat body cells. Thus, in our work, resistance to heat stress in males under diet with high carbohydrate content may contribute to increased fat body storage. Also, consumption of high carbohydrate diet might lead to nonenzymatic glycosylation and autooxidation monosaccharides, which might take part in ROS and RCS (reactive carbonyl species) production [8]. Therefore, ROS and RCS production could activate redox-sensitive transcriptional factors, including Forkhead box O (FOXO) transcription factors [38]. FOXO is involved in the upregulation of heat shock proteins (HSPs), which facilitates refolding and maintains proteostasis, thereby reducing the negative effects on the cell [39].

In *Drosophila*, dFOXO upregulates small heat shock proteins, including HSP22, HSP23, HSP26, and HSP27, [40] and large ones, including HSP70 [41]. In addition, the activated FOXO inhibits TOR activity, and consequently, it upregulates autophagy [42]. Thus, activated by ROS, dFOXO mediates autophagy and also upregulates Mn-SOD and catalase activities [43, 44]. Such effects may contribute to the maintenance of cellular homeostasis under oxidative stress.

We also demonstrated that flies of both sexes fed with a diet containing 1% of sucrose recovered quickly from chill coma. Such effects can also be related to HSP activation mediated by oxidative stress [39]. Furthermore, there are data, according to which, HSP induction occurs in response to cold stress [45]. Colinet and colleagues showed that *Hsp70Aa* and *Hsp68* were the most inducible during cold exposure in *Drosophila* strains. However, our results are controversial to previous works, which demonstrated enhanced resistance to cold stress under carbohydrate-enriched diet consumption [46].

Additional evidence regarding oxidative stress development in fruit flies under the consumption of diet with 1% of sucrose is the increased activity of antioxidant enzymes, including SOD in males and catalase in flies of both sexes. SOD and catalase are the first line antioxidant enzymes, which are responsible for superoxide anion and hydrogen peroxide detoxification, respectively [47]. Superoxide anion and other harmful radicals and nonradicals are produced in mitochondria, which seem to be the primary sources of ROS in the cell [48]. Oxidative stress is characterized by the increased abundance of oxidative damage of macromolecules, including proteins, lipids, and carbohydrates. Therefore, we measured the level of protein carbonyls, lipid peroxides, and protein- and low-molecular mass thiol groups as oxidative stress markers. In particular, the consumption of low sucrose diet caused increased levels of lipid peroxides and low-molecular mass thiols in females. Glutathione (GSH) represented over 80% of low-molecular mass thiols and played a pivotal role in resistance to oxidative damages [25]. This tripeptide is known to provide reducing equivalents for enzymes involved in the metabolism of ROS. Thus, GSH eliminates potentially toxic oxidation products and reduces oxidized biomolecules [27]. The antioxidant function of glutathione strongly depends on G6PDH and IDH activities, which are involved in the maintenance of NADPH pool for glutathione reduction. Our data showed increased activity of G6PDH and IDH in flies under low sucrose consumption. Obviously, low carbohydrate diet leads to oxidative stress development and antioxidant enzyme induction.

Additionally, we observed increased activity of GST in males that consumed the diet with 1% of sucrose. GST takes part in the detoxification of electrophilic compounds and toxic substrates [49]. Also, it is involved in the neutralization of the toxic carbonyl-, peroxide-, and epoxide-containing compounds, which are produced within the cell during oxidative stress by their conjugation with glutathione. GST might also be involved in the detoxification of the products of lipid peroxidation. It was shown that GST had an ability to neutralize 4‐hydroxynonenal (4‐HNE), which was one of the several end products of lipid peroxidation in the cells [50]. We found increased lipid peroxide levels in females that consumed the diet with 1% of sucrose. The GST activity in males may be increased to maintain the level of reduced glutathione and thus compensate the effects caused by a shift in the redox state [49, 51]. Moreover, the activity of GST also can provide resistance to cold stress exposure [52].

The increased activity of GST was also found in female flies fed with a diet containing 20% sucrose. In this case, GST induction could be associated with the increased level of protein carbonyls, content of which were elevated because of the consumption of 20% of sucrose. High carbohydrate consumption may induce such nonenzymatic processes like glycation [8]. The initiation of glycation takes place when a free carbonyl group of reducing sugar binds to a variety of macromolecules, mostly through the amino group of proteins and free amino acids [5]. This stage results in the formation of early glycated products, which are engaged in the generation of RCS [53]. Glycation is tightly connected with ROS formation as well [54]. Beyond that, it was investigated whether the antiglycation system reduced the RCS level by the activation of enzymes, such as amadoriases, carbonyl reductases, aldehyde, alcohol dehydrogenases, and GST, respectively [5]. It may explain the induction of GST and its relation to an elevated protein carbonyl level in flies of both sexes.

## 5. Conclusion

**Figure 6:**
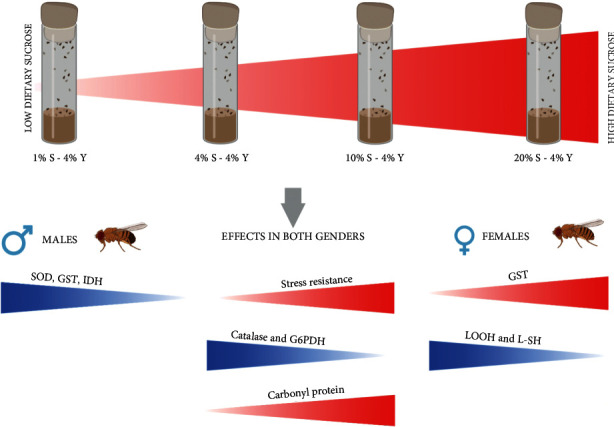
Effects of sucrose concentration in the diet on stress resistance, antioxidant enzymes activity, and oxidative stress markers in *D. melanogaster.*

These results strongly support the idea that dietary sugar content affects certain metabolic and oxidative processes. Increased survival under starvation, oxidative, heat, and cold stresses was observed by high sucrose concentration in the diet. Moreover, the increased levels of low-molecule mass thiols, lipid peroxides in females, and higher activity of antioxidant enzymes (Figure 6) indicate that the consumption of low carbohydrate diet could induce oxidative stress. We found that a high-carbohydrate diet results in increased protein carbonyls level, which may indicate the activation of glycation processes. Consequently, oxidative metabolism in *D. melanogaster* correlates with carbohydrate intake.

## Figures and Tables

**Table 1:** Two-way ANOVA of the impact of dietary sucrose concentration, sex, and their interaction on the investigated parameters.

Induces studied	Concentration, DF = 3		Sex, DF = 1		Concentration ^*∗*^ Sex, DF = 3	
	F ratio	*p*	F ratio	*p*	F ratio	*p*
SOD activity	11.62	<0.0001^*∗*^	34.94	<0.0001^*∗*^	8.718	0.0004^*∗*^
Catalase activity	9.977	0.0002^*∗*^	47.36	<0.0001^*∗*^	0.437	0.729
GST activity	2.929	0.054	0.008	0.930	8.961	0.0004^*∗*^
G6PDH activity	6.762	0.002^*∗*^	93.66	<0.0001^*∗*^	2.036	0.136
IDH activity	1.763	0.181	178.3	<0.0001^*∗*^	2.029	0.137
P-SH	0.538	0.663	100.5	<0.0001^*∗*^	0.346	0.793
L-SH	5.992	0.003^*∗*^	0.840	0.369	4.414	0.013^*∗*^
LOOH	12.00	0.0002^*∗*^	306.3	<0.0001^*∗*^	11.17	0.0003^*∗*^
Protein carbonyls	13.33	0.0001^*∗*^	16.40	0.0009^*∗*^	13.87	0.0001^*∗*^

^*∗*^Significant effect.

## Data Availability

The findings of this study are available from the corresponding author upon request.

## Abbreviations

SOD: Superoxide dismutase
ROS: Reactive oxygen species
GST: Glutathione-*S*-transferase
G6PDH: Glucose-6-phosphate dehydrogenase
IDH: Isocitrate dehydrogenase
HSP: Heat shock protein.
